# Determination of H5N1 Avian Influenza Virus Persistence Following a 2024 Backyard Poultry Outbreak in Romania

**DOI:** 10.3390/vetsci12100922

**Published:** 2025-09-23

**Authors:** Ionica Iancu, Florica Bărbuceanu, Emil Tîrziu, Corina Pascu, Luminița Costinar, Janos Degi, Corina Badea, Alexandru Gligor, Iulia Bucur, Sebastian Alexandru Popa, Maria Gurau, Viorel Herman

**Affiliations:** 1 Department of Infectious Diseases and Preventive Medicine, Faculty of Veterinary Medicine, University of Life Science “King Mihai I”, 300645 Timisoara, Romania; ionica.iancu@usvt.ro (I.I.); corinapascu@usvt.ro (C.P.); luminita.costinar@usvt.ro (L.C.); janosdegi@usvt.ro (J.D.); corina.badea@usvt.ro (C.B.); alexandru.gligor@usvt.ro (A.G.); viorelherman@usvt.ro (V.H.); 2 Faculty of Veterinary Medicine, University of Agronomical Sciences and Veterinary Medicine, 050097 Bucharest, Romania; maria.gurau@fmvb.usamv.ro; 3 Institute for Diagnosis and Animal Health (IDAH), 050557 Bucharest, Romania; 4 Department of Animal Production and Veterinary Public Health, Faculty of Veterinary Medicine, University of Life Science “King Mihai I”, 300645 Timisoara, Romania; emiltarziu@yahoo.com (E.T.); bucur_iulia@ymail.com (I.B.)

**Keywords:** H5N1, HPAI, virus persistence, backyard poultry, domestic waterfowl, RT-PCR, Romania, outbreak surveillance

## Abstract

In November 2024, an outbreak of highly pathogenic avian influenza (HPAI) H5N1 was confirmed in backyard poultry in Timiș County, Western Romania. The index cases involved chickens and domestic geese found dead with lesions characteristic of HPAI. Laboratory confirmation was achieved by real-time RT-qPCR targeting the matrix, H5, and N1 genes, followed by virus isolation in embryonated specific-pathogen-free eggs. Sequencing of the hemagglutinin cleavage site revealed the multi-basic motif PLREKRRKR/GLFG, consistent with a highly pathogenic phenotype. To investigate potential viral persistence, tracheal and cloacal swabs were collected from apparently healthy selected backyard poultry (chickens, geese, ducks and pheasants). RNA extraction and RT-qPCR, performed using protocols validated by the European Union Reference Laboratory for Avian Influenza, yielded negative results for all samples. Internal controls confirmed assay reliability, excluding the possibility of PCR inhibition. The investigation confirmed the occurrence of HPAI H5N1 in backyard poultry and demonstrated the absence of detectable viral persistence in surrounding flocks under the tested conditions. These findings highlight the importance of rapid molecular diagnostics, active surveillance, and strict biosecurity in limiting virus spread. Continued monitoring under the One Health framework is essential to mitigate the risk of avian influenza at the human–animal–environment interface.

## Simple Summary

In November 2024, a highly contagious bird disease called avian influenza (H5N1) was detected in backyard chickens and geese in western Romania. This virus often spreads quickly among birds and can cause sudden death. To confirm the cause, laboratory tests were carried out, including special genetic tests and virus isolation, which showed that the birds were infected with the highly dangerous form of the virus. Because such outbreaks can sometimes continue to spread silently, the study also tested apparently healthy chickens, ducks, geese, and pheasants in a nearby village. None of these birds carried the virus, suggesting that the outbreak was successfully contained and did not persist in the surrounding area. These results show the importance of quick testing, strict farm controls, and careful monitoring to stop further spread. They also highlight that small backyard flocks, when quickly managed, may not always lead to long-term virus circulation. This information is valuable for veterinarians, farmers, and public health authorities, as it supports rapid response strategies to protect both poultry health and human health.

## 1. Introduction

Highly pathogenic avian influenza (HPAI), caused by influenza A viruses of the H5 and H7 subtypes, remains a major threat to both poultry production and public health worldwide. In recent years, H5N1 viruses belonging to clade 2.3.4.4b have demonstrated unprecedented geographic expansion and host diversity, with incursions reported across Europe, Asia, Africa, and the Americas [1]. Clade 2.3.4.4b H5N1 has caused large-scale outbreaks globally, with transmission documented among wild birds, domestic poultry, and mammals, including recent cases in cattle and felines [2,3,4].

The virus exhibits substantial genetic diversity, characterized by multiple genotypes and mutations associated with adaptation to new host species [1,5,6,7,8]. The ecology of these viruses is increasingly complex, as it involves both migratory wild birds and domestic poultry. Migratory waterfowl often act as asymptomatic reservoirs and major vectors facilitating viral dissemination [1]. Active surveillance in high-risk areas such as wetlands and migratory corridors, combined with molecular testing of both domestic and wild birds, is crucial for outbreak control [8,9,10,11].

Recent studies also emphasize the importance of wildlife rehabilitation centers and live bird markets as critical points for monitoring viral circulation [11,12,13]. Expanding surveillance to mammals and other non-avian species has become increasingly relevant, given the observed cross-species spillover [11].

The adaptation of H5N1 to mammals elevates the zoonotic risk, with documented cases in humans, cattle, cats, seals, and sea lions. International guidelines (WOAH, WHO) highlight the urgent need for integrated surveillance at the human–animal–environment interface [5,6,14,15,16].

Romania has reported multiple HPAI outbreaks since the early 2000s, primarily in backyard poultry flocks located near wetlands and migratory bird corridors. In November 2024, an outbreak of HPAI H5N1 was detected in Timiș County, western Romania, affecting chickens and domestic geese raised in non-commercial holdings. The clinical presentation was characterized by sudden mortality and gross lesions indicative of avian influenza, which were subsequently confirmed through molecular diagnostics and virus isolation. Genetic characterization of the hemagglutinin cleavage site revealed the multi-basic motif PLREKRRKR/GLFG, consistent with a highly pathogenic phenotype. According to WOAH guidelines, post-outbreak surveillance is essential to confirm the absence of viral circulation and to support the recovery of disease-free status. Because viral persistence in the environment poses an epidemiological risk for secondary transmission to susceptible hosts, investigations were extended to adjacent rural areas.

Bethausen, a locality situated within the potential exposure zone of the original outbreak in Cutina (45.826382° N, 21.922722° E), was selected for targeted post-outbreak surveillance. Swabs from the trachea and cloaca of apparently healthy backyard chickens, geese, ducks, and pheasants were collected and subjected to validated molecular diagnostic testing. The objective of this investigation was to assess whether HPAI H5N1 could be detected in asymptomatic birds after the outbreak, thereby evaluating the potential for silent viral circulation within the surrounding area.

This work provides insight into the spatial containment of HPAI H5N1 outbreaks and underscores the role of targeted surveillance in small-scale domestic poultry settings. Furthermore, it highlights the importance of harmonized molecular diagnostics for early detection and control of avian influenza, in line with the One Health framework.

## 2. Materials and Methods

### 2.1. Outbreak Location and Sampling Strategy

Following the official notification of a highly pathogenic avian influenza (HPAI) outbreak in November 2024 in Cutina village (45.826382° N, 21.922722° E), Timiș County, western Romania, confirmatory sampling was performed by the county veterinary authorities under the coordination of the National Institute for Diagnosis and Animal Health (IDSA). The affected holdings were small-scale, non-commercial farms that housed mixed domestic bird species. Postmortem specimens were collected from dead chickens (*Gallus gallus domesticus*) and domestic geese (*Anser anser domesticus*), including brain, trachea, lungs, proventriculus, intestines, and liver.

**Figure 1:**
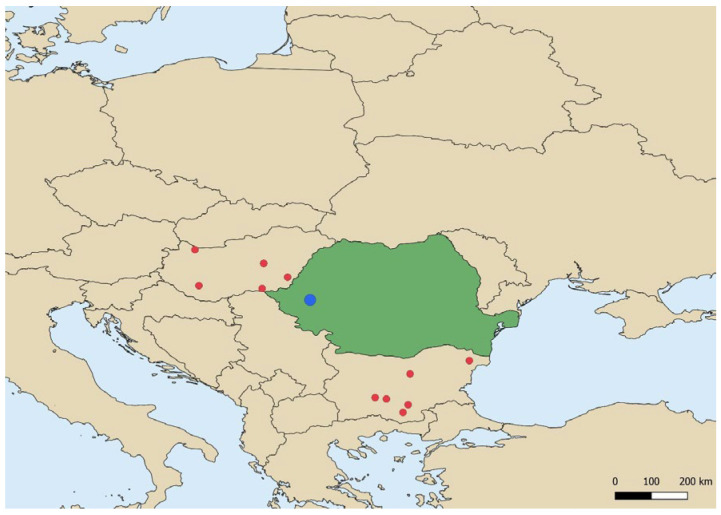
Geographic distribution of confirmed HPAI H5N1 outbreaks in Central and Southern Europe during the 2023–2024 season. Red dots indicate outbreaks reported in the region, while the blue dots marks the Romanian outbreak site in Timiș county.

To investigate potential viral persistence beyond the outbreak site in Cutina (45.826382° N, 21.922722° E—Figure 1), additional sampling was conducted in Bethausen (approx. 45.8889° N, 21.8833° E), a locality situated within the 10 km surveillance zone. Tracheal and cloacal swabs were collected from apparently healthy selected backyard poultry (chickens, geese, ducks and pheasants). They were aseptically collected from each bird and transported to the IDSA laboratory under refrigerated conditions (4–6 °C), in accordance with WOAH recommendations for avian influenza surveillance [17]. Sampling was carried out during the 30-day post-outbreak surveillance period, in compliance with EU Regulation 2020/687 and WOAH recommendations [17,18].

Due to the emergency response context of the outbreak and the limited time window for post-outbreak surveillance, the selection of birds for persistence testing was performed opportunistically, based on owner availability and logistical feasibility. No formal randomization was applied. Sampling was coordinated by county veterinary authorities, targeting backyard holdings located within the surveillance zone. This approach aligns with WOAH guidelines for field-based outbreak response, where convenience sampling may be appropriate to demonstrate the absence of ongoing viral circulation rather than estimate prevalence.

This investigation was conducted in response to a naturally occurring HPAI H5N1 outbreak rather than under controlled experimental conditions. As a result, sample selection, timing, and study scope were constrained by the realities of field epidemiology and emergency veterinary response.

The collection of samples was carried out by the official veterinary authorities in accordance with national regulations, in particular with the Veterinary Orders no. 145/2018 and 36/2010, which establish standardized procedures for the surveillance, notification and taking samples from animals suspected of diseases with mandatory declaration [19,20].

### 2.2. Sample Preparation and RNA Extraction

Organ samples from the outbreak site were mechanically homogenized using ceramic bead tubes, and tissue supernatants were pooled according to organ system before RNA extraction. Total RNA was extracted with the Total RNA Purification Kit (Analytik Jena, Germany), following the manufacturer’s instructions for tissue matrices. For the persistence investigation, swab samples were hydrated in antibiotic-supplemented medium, as recommended by the WOAH Manual for avian influenza [17].

Tracheal swabs were suspended in 1 mL of medium, whereas cloacal swabs were diluted in 2 mL to reduce the effect of potential PCR inhibitors.

RNA extraction from swab suspensions was performed using the IndiMag Pathogen Kit (Indical Bioscience, Leipzig, Germany), with the Type^®^ RNA internal control included to monitor for inhibitory substances and to verify extraction efficiency. The protocol ensured RNase inactivation, DNase treatment for the elimination of genomic DNA, and elution in RNase-free molecular-grade water, following validated procedures recommended by the European Union Reference Laboratory for Avian Influenza (EURL, IZSVe, Legnaro, Italy) [21,22].

### 2.3. Molecular Detection by Real-Time RT-PCR

All samples were tested for influenza A virus RNA using a validated real-time reverse transcription PCR (RT-qPCR) targeting the matrix (M) gene. The oligonucleotides used for the influenza A virus matrix assay were: sense primer (IVA D161M): AGA TGA GYC TTC TAA CCG AGG TCG; antisense primer (IVA D162M): TGC AAA NAC ATC YTC AAG TCT CTG; and probe (IVA MA): FAM–TCA GGC CCC CTC AAA GCC GA–TAMRA/BHQ, as previously described [21,23]. Primers for the M gene were originally reported by Van der Goot et al. [23], and the assay design was further validated by Heine et al. [21] and included in the EURL diagnostic protocols [22].

Subtyping assays were then performed on the same RNA extracts. The oligonucleotide sequences for identification of the H5 subtype were based on the validated RT-qPCR assay described by Slomka et al. [24], which targets the HA2 subunit of Eurasian lineage H5 viruses. The identification of the neuraminidase N1 subtype was conducted according to the protocol of Hassan et al. [25], using primers located in a highly conserved region of segment 6 of Eurasian neuraminidases.

Amplification reactions were carried out using the AgPath-ID™ One-Step RT-PCR Kit (Thermo Fisher Scientific, Waltham, MA, USA) on QuantStudio™ 7 Flex and LightCycler^®^ 480 platforms, in accordance with EURL and WOAH recommendations [17,18]. An enhanced green fluorescent protein (EGFP) internal control reaction, with primers and a Cy5-labeled probe, was included in all runs to confirm amplification validity. The consistent Ct values of the EGFP control across all reactions confirmed the absence of PCR inhibition and supported the reliability of the negative results obtained for the matrix gene target.

### 2.4. Subtype Identification and Sequencing

Samples that tested positive for influenza A virus RNA underwent subtype determination for H5, H7, and N1 genes using WOAH-validated RT-qPCR assays [17,22]. Subtyping was performed on the same RNA extracts using primer–probe sets targeting conserved regions of the hemagglutinin (HA) and neuraminidase (NA) genes, following EURL recommendations [22]. Positive results for H5 and N1 were subsequently confirmed by virus isolation in embryonated specific-pathogen-free chicken eggs, according to WOAH procedures [17].

For molecular characterization, the hemagglutinin cleavage site was amplified by RT-PCR and sequenced using the Sanger method. Amino acid sequences were aligned and analyzed to assess pathogenicity. Due to the emergency context, genomic determinations were externalized to the European Union Reference Laboratory for Avian Influenza (IZSVe, Legnaro, Italy), where sequencing of the hemagglutinin cleavage site was performed. The presence of a multi-basic cleavage site motif (PLREKRRKR/GLFG) was considered indicative of a highly pathogenic avian influenza virus (HPAIV), in accordance with international classification criteria [17,26]. Phylogenetic comparison placed the Romanian outbreak strains within clade 2.3.4.4b, consistent with viruses circulating across Europe during the 2023–2024 season [27].

### 2.5. Phylogenetic Analysis

Complete coding sequences of the hemagglutinin (HA) gene from the two Romanian H5N1 isolates generated in this study (A/laying-hen/Romania/15681_25VIR1011-4/2024, EPI_ISL_19725315; and A/domestic-goose/Romania/15682_25VIR1011-5/2024, EPI_ISL_19725316) were analyzed together with representative clade 2.3.4.4b H5N1 strains previously reported from Serbia (e.g., A/Common crane/Serbia/12661/2023, GenBank accession numbers PQ685008–PQ685015) and Bulgaria (GenBank accession numbers OQ664048–OQ664055).

Multiple sequence alignment was performed using MAFFT v7.511 with default parameters, followed by manual inspection to ensure codon accuracy. Poorly aligned positions and terminal gaps were removed using TrimAl v1.4 to improve the phylogenetic signal. The curated alignment was then used to infer a maximum likelihood (ML) phylogenetic tree with IQ-TREE v2.3.5, applying the best-fit nucleotide substitution model selected automatically by ModelFinder according to the Bayesian Information Criterion (BIC). Branch support was assessed with 1000 ultrafast bootstrap replicates combined with SH-aLRT (Shimodaira–Hasegawa approximate likelihood ratio test) to provide robust confidence estimates for internal nodes.

An H5N8 reference strain was used as an outgroup to root the phylogeny and facilitate clade interpretation. The resulting ML tree was visualized and annotated using iTOL v7 and Phylo.io (https://phylo.io/), and exported in high-resolution SVG and PNG formats for inclusion in the manuscript. Romanian isolates were highlighted to illustrate their genetic relatedness within the European clade 2.3.4.4b context.

## 3. Results

### 3.1. HPAI H5N1 Confirmation in Outbreak Samples

All organ samples collected from dead chickens (*Gallus gallus domesticus*, n = 8) and domestic geese (*Anser anser domesticus*, n = 2) in Cutina tested positive for avian influenza A virus by RT-qPCR targeting the matrix gene. Subtyping assays confirmed the presence of H5 and N1 genes in all cases, while H7 was not detected.

In addition to molecular confirmation, postmortem examination of the dead chickens and geese revealed gross lesions characteristic of HPAI. The most consistent findings included multifocal petechial and ecchymotic hemorrhages on the epicardium, proventricular serosa, and intestinal mucosa, along with severe pulmonary congestion and edema. Hemorrhagic tracheitis and pancreatitis were also noted in several birds. In geese, marked enteritis with mucosal hemorrhage was observed, while in chickens, congestion and small hemorrhages were present in the brain, suggestive of neurologic involvement. These lesions are in line with the classical pathological presentation of highly pathogenic avian influenza and support the virological and molecular results obtained.

Successful virus isolation was achieved in embryonated SPF chicken eggs for all tested samples, in accordance with WOAH guidelines [17] and EURL diagnostic protocols [22]. Sequencing of the hemagglutinin gene revealed the multibasic cleavage site motif PLREKRRKR/GLFG, characteristic of highly pathogenic avian influenza viruses. This molecular feature confirmed the etiological agent as HPAI H5N1, consistent with viruses of clade 2.3.4.4b circulating in Europe during the same season [26,27]. A summary of the molecular and virological results obtained from the outbreak site is presented in Table 1, which documents the detection of influenza A virus genes, successful virus isolation, and pathogenic cleavage site characterization.

**Figure 2:**
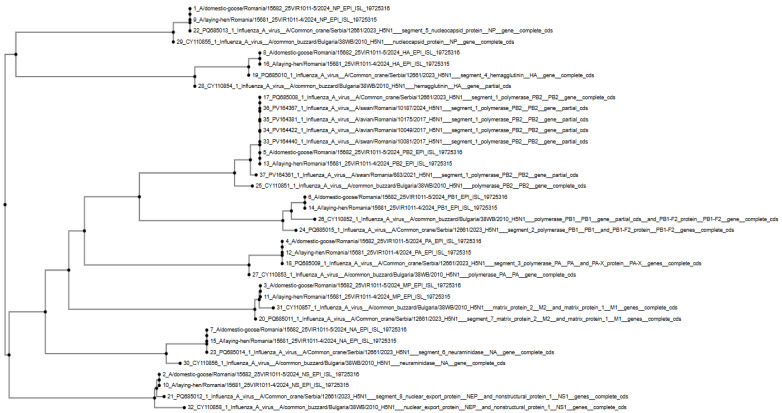
Maximum-likelihood phylogenetic tree of H5N1 clade 2.3.4.4b influenza A viruses isolated from domestic birds in Romania (2024) and representative strains from neighboring European countries (Serbia, Bulgaria).

A phylogenetic analysis based on HA gene sequences confirmed that the Romanian outbreak isolates clustered within clade 2.3.4.4b together with closely related strains from Serbia and Bulgaria, indicating a common evolutionary origin (Figure 2).

The phylogenetic tree was constructed based on complete or partial coding sequences of multiple genomic segments (PB2, PB1, PA, NP, HA, NA, M, NS). Sequences from Romanian isolates (EPI_ISL_19725316 A/domestic-goose/Romania/15682_25VIR1011-5/2024 and EPI_ISL_19725315 A/laying-hen/Romania/15681_25VIR1011-4/2024) were aligned with representative H5N1 clade 2.3.4.4b sequences from Serbia (GenBank accessions PQ685008–PQ685015) and Bulgaria (e.g., CY110855, CY110858, CY110854, CY110851). The alignment was performed using MAFFT (v7) and trimmed with TrimAl to remove poorly aligned regions. The phylogenetic tree was inferred using the maximum-likelihood method implemented in IQ-TREE (v2.3) under the GTR + G substitution model with 1000 ultrafast bootstrap replicates and SH-aLRT support values. Black dots on branches indicate nodes with both bootstrap and SH-aLRT support ≥ 90%. The scale bar represents the number of nucleotide substitutions per site.

### 3.2. Absence of AIV Detection in Persistence Study Samples

As part of the post-outbreak surveillance, 240 apparently healthy backyard birds (sixty chickens, sixty ducks, sixty geese, and sixty pheasants) were sampled within the 10 km surveillance zone. A total of 480 tracheal and cloacal swabs tested negative for influenza A virus RNA by RT-qPCR targeting the matrix gene. Internal control amplification was consistent, confirming the reliability of negative results.

These findings indicate that no evidence of HPAI H5N1 persistence or asymptomatic circulation was detected in backyard poultry flocks within the surveillance zone at the time of investigation. Multiple studies indicate that while HPAI H5N1 can infect backyard poultry, persistent or asymptomatic circulation is rarely detected in well-conducted surveillance zones. For example, cross-sectional studies in Bangladesh found no active H5N1 virus in backyard chickens by RT-PCR, though some birds had H5 antibodies, suggesting past exposure rather than ongoing infection [28]. In Indonesia, H5N1 is considered endemic in backyard poultry, but persistence is linked to poor biosecurity and frequent contact with wild birds, not to silent, ongoing circulation within isolated flocks [29,30]. In Europe, outbreaks in backyard flocks are typically associated with clear clinical signs and mortality, not asymptomatic persistence [27].

The results of the present investigation align with WOAH recommendations, which emphasize that early implementation of containment measures and the structural separation of smallholder farms can effectively reduce the risk of local viral persistence [17].

## 4. Discussion

The present study reports the confirmation and molecular characterization of a highly pathogenic avian influenza (HPAI) H5N1 outbreak in backyard poultry in western Romania and evaluates the potential persistence of the virus in neighboring rural areas. The etiological agent was confirmed as H5N1, with sequencing of the hemagglutinin (HA) cleavage site revealing the multi-basic motif PLREKRRKR/GLFG, a recognized molecular marker of high pathogenicity in avian influenza viruses [17,26]. This motif is consistent with viruses of clade 2.3.4.4b that circulated widely across Europe during the 2023–2024 winter season, frequently associated with wild bird migration pathways. These findings align with the broader epidemiological trend observed across Europe during the same period, in which HPAI outbreaks were repeatedly reported in both wild and domestic birds.

Previous investigations, together with broader European surveillance data, demonstrate that HPAI H5N1 outbreaks are strongly associated with migratory bird movements, with Romania and neighboring countries repeatedly experiencing introductions along major flyways that connect regional wetlands to wider continental routes [31,32,33].

All these viruses harbored a multi-basic cleavage site motif (PLREKRRKR/GLF), confirming their highly pathogenic phenotype. Global studies further support that Europe remains a high-risk region for HPAI H5N1 outbreaks, characterized by seasonal recurrence and a clear association with bird density and migratory flyways [34].

Importantly, the follow-up investigation targeting viral persistence in birds from Bethausen, a locality neighboring the outbreak site, yielded no positive results. All samples collected from apparently healthy chickens, ducks, geese, and pheasants tested negative for AIV RNA. The presence of robust amplification in the internal controls excluded the possibility of technical errors or PCR inhibition, reinforcing the conclusion that no viral circulation was ongoing at the time of testing.

Extensive European surveillance reports confirm that virological testing of apparently healthy birds within outbreak zones is a key strategy to exclude ongoing or subclinical HPAI circulation, as recommended by WOAH and EU Regulation 2020/687 [35,36,37,38,39,40].

Multiple EFSA overviews document that, following outbreaks, targeted sampling of healthy chickens, ducks, geese, and other species within the surveillance window (typically 30 days) consistently yields negative results for AIV RNA when no further cases are present, supporting the sufficiency of a single round of virological testing combined with passive surveillance [35,36,37,38,39,40].

According to EU Regulation 2020/687, one round of virological sampling within the 30-day post-outbreak monitoring period, combined with passive surveillance for clinical signs and mortality, is considered sufficient to demonstrate the absence of viral persistence when no further cases are reported.

The swift implementation of control measures, together with the inherent structural separation of backyard farm [35,36,40], characterized by small flock sizes and limited inter-farm contact, appears to have been sufficient to halt further spread and effectively contain the outbreak [36,37,38,40].

In addition, the lack of asymptomatic shedding in waterfowl and pheasants suggests that, under certain rural conditions, localized outbreaks may not persist in the environment, especially where bird density is low and inter-farm contact is limited.

Nevertheless, some limitations of the present study should be acknowledged. Surveillance reports acknowledge that limited sample sizes, reliance solely on molecular detection, and single time-point sampling may miss low-level or transient viral shedding [38,41].

Complementary serological testing and environmental sampling would have provided additional insights into subclinical exposure and viral shedding. Moreover, the single-time-point sampling design did not allow the assessment of temporal dynamics in viral persistence.

These findings underscore the necessity for ongoing surveillance, particularly in areas where small-scale poultry farming overlaps with wild bird habitats. The integration of molecular diagnostics with epidemiological monitoring is essential for early detection and for guiding rapid containment strategies. The use of validated protocols and internal controls, as demonstrated in this study, enhances diagnostic reliability and confidence in negative results.

The confirmed outbreak of HPAI H5N1 in Timiș County, Romania, occurred within the broader context of continued virus circulation across Europe during the 2023–2024 season. Data reported to the WOAH and summarized by EFSA indicate that, between December 2023 and March 2024, outbreaks of HPAI H5N1 in domestic and wild birds were reported in at least 26 European countries, including Hungary, Bulgaria, Poland, Germany, and Italy [39,40,42].

Most outbreaks in domestic poultry were primary incursions, linked to virus introduction by migratory wild birds, with high-risk zones corresponding to migratory corridors such as the Pannonian Basin, located near Romania’s western border [40,41,43].

Genetic characterization further confirmed that Romanian H5N1 isolates harbored the multi-basic cleavage site motif PLREKRRKR/GLFG, identical to sequences detected in viruses from Hungary and Serbia at the end of 2024. These findings suggest potential regional virus flow or shared migratory sources. Circulation of clade 2.3.4.4b was dominant in Europe during this period, with the emergence of several novel reassorting genotypes, while maintaining genetic similarities between outbreaks in neighboring countries [40,41,44].

The Pannonian Basin is recognized as a high-risk area for avian influenza incursion, and outbreaks in western Romania, approximately 80 km from the Hungarian frontier, fall within this elevated-risk context. Spatiotemporal studies confirm that Central and Eastern Europe remain hotspots for HPAI H5N1 circulation, with predominant transmission along the northwest–southeast axis, shaped by wild bird migration routes and poultry population density [34,40].

In contrast to larger commercial outbreaks reported in Central Europe, the Romanian case was restricted to backyard flocks, with rapid containment and no documented spillover to wild birds. Moreover, no evidence of viral persistence was detected in surrounding areas, differing from observations in Hungary and Germany, where recurrent detections in wild avifauna were reported weeks after primary poultry outbreaks. Germany and Hungary have reported numerous outbreaks of HPAI H5N1 in both domestic and wild birds in recent years, with persistent virus circulation and the emergence of novel genotypes, particularly within clade 2.3.4.4b [35,36,37,38].

During the 2021–2022 season, Germany reported the highest number of detections in wild birds, while Hungary recorded the second-highest number of domestic outbreaks, most of which occurred in commercial duck and goose farms [35,36,45].

Genetic analyses demonstrate that viruses from Germany and Hungary are closely related to those detected in other European countries, with multiple reassortment events and introductions attributed to wild bird migration. Virus persistence in wild bird populations and the high density of commercial farms are major risk factors for HPAI H5N1 spread in both countries [37,38,39].

In Hungary, most outbreaks were secondary, resulting from farm-to-farm transmission rather than repeated introductions from wild birds [46]. These differences support the hypothesis that smallholder outbreaks, if promptly diagnosed and effectively contained, may pose a lower risk of prolonged environmental contamination compared to outbreaks in high-density commercial operations.

The persistence investigation confirmed the absence of influenza A virus RNA in apparently healthy backyard poultry sampled in a neighboring locality. The inclusion of internal amplification controls excluded the possibility of false negatives, thereby reinforcing the validity of the results [17].

Comparison with contemporaneous European outbreaks highlights notable differences in outbreak dynamics. Several countries, including Hungary, Germany, Poland, and Italy, reported recurrent HPAI H5N1 detections in both domestic poultry and wild birds [35,36,37,38,40,47,48].

By contrast, the Romanian outbreak remained geographically restricted to backyard holdings, with no confirmed spillover into wild avifauna or neighboring domestic flocks. Furthermore, while persistent detection of HPAI in wild birds was reported in Central and Western Europe, no viral persistence was identified in the present study area. This contrast underscores the influence of farming systems, biosecurity measures, and ecological context in shaping outbreak outcomes.

Interpretation of these results requires caution, as the persistence investigation was constrained by a relatively limited sample size (240 birds) and a single time-point design, while the exclusive reliance on molecular detection precluded the identification of prior exposures or transient low-prevalence infections that might otherwise have been revealed through serological or longitudinal approaches.

Despite these constraints, the study offers valuable epidemiological insights. The results emphasize the importance of rapid laboratory diagnosis, validated molecular methods, and harmonized surveillance protocols for effective outbreak containment. From a broader perspective, these findings highlight the relevance of a One Health framework, given the zoonotic potential of HPAI H5N1 and its ability to cross species barriers. Strengthening surveillance in regions where backyard poultry overlaps with wild bird habitats remains essential for early detection and prevention of further spread.

## 5. Conclusions

The present study confirmed the presence of HPAI H5N1 in backyard poultry in western Romania and demonstrated the absence of viral persistence in neighboring flocks under the tested conditions. The identified strain belonged to clade 2.3.4.4b and carried the multi-basic hemagglutinin cleavage site motif PLREKRRKR/GLFG, a molecular hallmark of high pathogenicity. The absence of virus detection in apparently healthy poultry from a nearby locality indicates that implemented control measures and the structural separation of smallholder farms were effective in preventing further spread.

Our findings underscore the strength of validated molecular diagnostics and harmonized surveillance as essential tools for rapid outbreak containment. Despite certain limitations, the study provides valuable epidemiological evidence on the dynamics of localized outbreaks and demonstrates the effectiveness of prompt interventions in small-scale poultry systems.

From a wider perspective, the results highlight the critical importance of sustained surveillance at the interface between backyard poultry and wild bird habitats. Adopting a One Health approach remains fundamental for mitigating avian influenza risks and protecting both animal and public health.

## Figures and Tables

**Table 1:** Molecular confirmation of HPAI H5N1 in backyard poultry, Cutina (Timiș, Romania)—Nov 2024.

Sample ID	Species	Matrix Gene	H5	N1	H7	Virus Isolation	HA Cleavage Site	Pathotype
16890-1	Chicken	Positive	+	+	–	Positive	PLREKRRKR/GLFG	HPAI
16890-2	Chicken	Positive	+	+	–	Positive	PLREKRRKR/GLFG	HPAI
16890-3	Chicken	Positive	+	+	–	Positive	PLREKRRKR/GLFG	HPAI
16890-4	Chicken	Positive	+	+	–	Positive	PLREKRRKR/GLFG	HPAI
16890-5	Chicken	Positive	+	+	–	Positive	PLREKRRKR/GLFG	HPAI
16890-6	Chicken	Positive	+	+	–	Positive	PLREKRRKR/GLFG	HPAI
16890-7	Chicken	Positive	+	+	–	Positive	PLREKRRKR/GLFG	HPAI
16890-8	Chicken	Positive	+	+	–	Positive	PLREKRRKR/GLFG	HPAI
16889-1	Domestic goose	Positive	+	+	–	Positive	PLREKRRKR/GLFG	HPAI
16889-2	Domestic goose	Positive	+	+	–	Positive	PLREKRRKR/GLFG	HPAI

## Data Availability

The original contributions presented in this study are included in the article. Further inquiries can be directed to the corresponding author.

## References

[1] Xie Z., Yang J., Jiao W., Li X., Iqbal M., Liao M., Dai M. (2025). Clade 2.3.4.4b Highly Pathogenic Avian Influenza H5N1 Viruses: Knowns, Unknowns, and Challenges. J. Virol..

[2] Webby R., Uyeki T. (2024). An Update on Highly Pathogenic Avian Influenza A(H5N1) Virus, Clade 2.3.4.4b. J. Infect. Dis..

[3] Burrough E., Magstadt D., Petersen B., Timmermans S., Gauger P., Zhang J., Siepker C., Mainenti M., Li G., Thompson A. (2024). Highly Pathogenic Avian Influenza A(H5N1) Clade 2.3.4.4b Virus Infection in Domestic Dairy Cattle and Cats, United States, 2024. Emerg. Infect. Dis..

[4] Li Y., Sun Z., Liu X., Wei S., Zhang Y., Fuxiang Y., Qiao J., Zhang H., Xiao C. (2024). Highly Pathogenic Avian Influenza A(H5N1) Virus Infection in Dairy Cattle: Threat of Bird Flu Has Expanded to Open-Air Farmed Livestock. J. Infect..

[5] Steinsiepe V., Cruz C., Icochea M., Espejo V., Troncos G., Castro-Sanguinetti G., Schilling M., Tinoco Y. (2023). Highly Pathogenic Avian Influenza A(H5N1) from Wild Birds, Poultry, and Mammals, Peru. Emerg. Infect. Dis..

[6] Rimondi A., Vanstreels R., Olivera V., Donini A., Lauriente M., Uhart M. (2024). Highly Pathogenic Avian Influenza A(H5N1) Viruses from Multispecies Outbreak, Argentina, August 2023. Emerg. Infect. Dis..

[7] Tian J., Bai X., Li M., Zeng X., Xu J., Li P., Wang M., Song X., Zhao Z., Tian G. (2023). Highly Pathogenic Avian Influenza Virus (H5N1) Clade 2.3.4.4b Introduced by Wild Birds, China, 2021. Emerg. Infect. Dis..

[8] Leguía M., Garcia-Glaessner A., Muñoz-Saavedra B., Juárez D., Barrera P., Calvo-Mac C., Jara J., Silva W., Ploog K., Amaro L. (2023). Highly Pathogenic Avian Influenza A (H5N1) in Marine Mammals and Seabirds in Peru. Nat. Commun..

[9] Ariyama N., Pardo-Roa C., Muñoz G., Aguayo C., Ávila C., Mathieu C., Almonacid L., Medina R., Brito B., Johow M. (2023). Highly Pathogenic Avian Influenza A(H5N1) Clade 2.3.4.4b Virus in Wild Birds, Chile. Emerg. Infect. Dis..

[10] Bevins S., Shriner S., Cumbee J., Dilione K., Douglass K., Ellis J., Killian M., Torchetti M., Lenoch J. (2022). Intercontinental Movement of Highly Pathogenic Avian Influenza A(H5N1) Clade 2.3.4.4 Virus to the United States, 2021. Emerg. Infect. Dis..

[11] El-Shesheny R., Moatasim Y., Mahmoud S., Song Y., Taweel A., Gomaa M., Kamel M., Sayes M., Kandeil A., Lam T. (2022). Highly Pathogenic Avian Influenza A(H5N1) Virus Clade 2.3.4.4b in Wild Birds and Live Bird Markets, Egypt. Pathogens.

[12] Hall V., Cardona C., Mendoza K., Torchetti M., Lantz K., Bueno I., Franzen-Klein D. (2024). Surveillance for Highly Pathogenic Avian Influenza A (H5N1) in a Raptor Rehabilitation Center—2022. PLoS ONE.

[13] Jimenez-Bluhm P., Siegers J., Tan S., Sharp B., Freiden P., Johow M., Orozco K., Ruiz S., Baumberger C., Galdames P. (2023). Detection and Phylogenetic Analysis of Highly Pathogenic A/H5N1 Avian Influenza Clade 2.3.4.4b Virus in Chile, 2022. Emerg. Microbes Infect..

[14] Puryear W., Sawatzki K., Hill N., Foss A., Stone J., Doughty L., Walk D., Gilbert K., Murray M., Cox E. (2023). Highly Pathogenic Avian Influenza A(H5N1) Virus Outbreak in New England Seals, United States. Emerg. Infect. Dis..

[15] Briand F., Souchaud F., Pierre I., Beven V., Hirchaud E., Hérault F., Planel R., Rigaudeau A., Bernard-Stoecklin S., Van Der Werf S. (2023). Highly Pathogenic Avian Influenza A(H5N1) Clade 2.3.4.4b Virus in Domestic Cat, France, 2022. Emerg. Infect. Dis..

[16] Thorsson E., Zohari S., Roos A., Banihashem F., Bröjer C., Neimanis A. (2023). Highly Pathogenic Avian Influenza A(H5N1) Virus in a Harbor Porpoise, Sweden. Emerg. Infect. Dis..

[17] WOAH (2021). Avian Influenza (Infection with Avian Influenza Viruses). Manual of Diagnostic Tests and Vaccines for Terrestrial Animals.

[18] European Union (2020). Commission Implementing Regulation (EU) 2020/687 of 17 December 2019 Laying Down Rules for the Application of Regulation (EU) 2016/429 of the European Parliament and the Council as Regards the Prevention and Control of Certain Listed Diseases. Off. J. Eur. Union.

[19] Autoritatea Națională Sanitară Veterinară și pentru Siguranța Alimentelor (ANSVSA) Ordinul Nr. 145/2018 pentru Aprobarea Normei Sanitare Veterinare Privind Supravegherea, Prevenirea, Controlul și Eradicarea Gripei Aviare. Monitorul Oficial al României 2018, Partea I, Nr. 642/24.VII.2018. https://legislatie.just.ro/Public/DetaliiDocument/207313.

[20] Autoritatea Națională Sanitară Veterinară și pentru Siguranța Alimentelor (ANSVSA) Ordinul Nr. 36/2010 pentru Aprobarea Normei Sanitare Veterinare Privind Notificarea și Raportarea Bolilor la Animale. Monitorul Oficial al României 2010, Partea I, Nr. 223/09.IV.2010. https://legislatie.just.ro/public/DetaliiDocument/118165.

[21] Heine H.G., Foord A.J., Wang J., Valdeter S., Walker S., Morrissy C., Wong F.Y., Meehan B. (2015). Detection of Highly Pathogenic Zoonotic Influenza Virus H5N6 by Reverse-Transcriptase Quantitative Polymerase Chain Reaction. Virol. J..

[22] EURL (European Union Reference Laboratory for Avian Influenza) (2022). EURL Diagnostic Protocols for Avian Influenza Virus Detection and Subtyping.

[23] Van der Goot J.A., Koch G., De Jong M.C., Van Boven M. (2005). Transmission of Highly Pathogenic Avian Influenza H5N1 Virus in Pekin Ducks Is Significantly Reduced by a Genetically Distant H5N2 Vaccine. Virology.

[24] Slomka M.J., Coward V.J., Banks J., Löndt B.Z., Brown I.H., Voermans J., Koch G., Handberg K.J., Jørgensen P.H., Cherbonnel-Pansart M. (2007). Validated H5 Eurasian Real-Time Reverse Transcriptase Polymerase Chain Reaction and Its Application in H5N1 Outbreaks in 2005–2006. Avian Dis..

[25] Hassan K.E., Balz K., Tuppurainen E., El Zowalaty M.E., Ulrich R., Beer M., Hoffmann B. (2022). Improved Subtyping of Avian Influenza Viruses Using an RT-qPCR Based Low Density Array: Riems Influenza A Typing Array (RITA 2). Viruses.

[26] Lee D.H., Bertran K., Kwon J.H., Swayne D.E. (2017). Evolution, Global Spread, and Pathogenicity of Highly Pathogenic Avian Influenza H5Nx Clade 2.3.4.4. J. Vet. Sci..

[27] Adlhoch C., Fusaro A., Kuiken T., Niqueux É., Staubach C., Terregino C., Aznar I., Guajardo I.M., Baldinelli F. (2023). Avian Influenza Overview December 2022–March 2023. EFSA J..

[28] Gupta S., Hoque M., Fournié G., Henning J. (2020). Patterns of Avian Influenza A (H5) and A (H9) Virus Infection in Backyard, Commercial Broiler and Layer Chicken Farms in Bangladesh. Transbound. Emerg. Dis..

[29] Rehman S., Effendi M., Witaningruma A., Nnabuike U., Bilal M., Abbas A., Abbas R., Hussain K. (2022). Avian Influenza (H5N1) Virus, Epidemiology and Its Effects on Backyard Poultry in Indonesia: A Review. F1000Research.

[30] Hidayat M., Dewi A., Schoonman L., Wibawa H., Lubis E., Lockhart C., Setiaji G., McGrane J., Vink W. (2020). Investigating the Endemic Presence and Persistence of HPAI H5N1 Virus on Java, Indonesia. Authorea.

[31] Ward M., Maftei D., Apostu C., Suru A. (2009). Association between outbreaks of highly pathogenic avian influenza subtype H5N1 and migratory waterfowl (family Anatidae) populations. Zoonoses Public Health.

[32] Stoimenov G., Goujgoulova G., Hristov K. (2020). Analysis of a highly pathogenic avian influenza (H5N1) virus causing the first outbreak in domestic poultry in Bulgaria in January 2015. Vet. Med..

[33] Lewis N., Banyard A., Whittard E., Karibayev T., Kafagi T., Chvala I., Byrne A., Akberovna S., King J., Harder T. (2021). Emergence and spread of novel H5N8, H5N5 and H5N1 clade 2.3.4.4 highly pathogenic avian influenza in 2020. Emerg. Microbes Infect..

[34] Li Y., An Q., Sun Z., Gao X., Wang H. (2024). Multifaceted analysis of temporal and spatial distribution and risk factors of global poultry HPAI-H5N1, 2005–2023. Animals.

[35] Adlhoch C., Fusaro A., Gonzáles J., Kuiken T., Marangon S., Niqueux E., Staubach C., Terregino C., Aznar I., Guajardo I. (2022). Avian influenza overview May–September 2021. EFSA J..

[36] Adlhoch C., Fusaro A., Gonzáles J., Kuiken T., Marangon S., Niqueux E., Staubach C., Terregino C., Aznar I., Guajardo I. (2021). Avian influenza overview September–December 2021. EFSA J..

[37] Adlhoch C., Fusaro A., Gonzáles J., Kuiken T., Marangon S., Niqueux E., Staubach C., Terregino C., Aznar I., Guajardo I. (2022). Avian influenza overview March–June 2022. EFSA J..

[38] Adlhoch C., Fusaro A., Gonzáles J., Kuiken T., Marangon S., Niqueux E., Staubach C., Terregino C., Aznar I., Guajardo I. (2022). Avian influenza overview December 2021–March 2022. EFSA J..

[39] Adlhoch C., Fusaro A., Gonzáles J., Kuiken T., Marangon S., Niqueux E., Staubach C., Terregino C., Guajardo I., Chuzhakina K. (2022). Avian influenza overview June–September 2022. EFSA J..

[40] Fusaro A., Gonzales J., Kuiken T., Mirinaviciute G., Niqueux E., Ståhl K., Staubach C., Svartström O., Terregino C., Willgert K. (2024). Avian influenza overview December 2023–March 2024. EFSA J..

[41] Verhagen J., Fouchier R., Lewis N. (2021). Highly pathogenic avian influenza viruses at the wild–domestic bird interface in Europe: Future directions for research and surveillance. Viruses.

[42] Gurău M.R., Oțelea F., Negru E., Șonea C., Beșleagă S., Bărbucianu F., Herman V., Iancu I., Baraitareanu S., Daneș D. (2024). PCR method and Sanger sequencing for PB2 fragment detection in influenza virus strains from Romania. Rev. Rom. Med. Vet..

[43] Adlhoch C., Fusaro A., Gonzales J., Kuiken T., Mirinaviciute G., Niqueux E., Ståhl K., Staubach C., Terregino C., Willgert K. (2023). Avian influenza overview September–December 2023. EFSA J..

[44] Van Borm S., Ahrens A., Bachofen C., Banyard A., Bøe C., Briand F., Dirbáková Z., Engelsma M., Fusaro A., Germeraad E. (2025). Genesis and spread of novel highly pathogenic avian influenza A(H5N1) clade 2.3.4.4b virus genotype EA-2023-DG reassortant, Western Europe. Emerg. Infect. Dis..

[45] King J., Harder T., Globig A., Stacker L., Günther A., Grund C., Beer M., Pohlmann A. (2022). Highly pathogenic avian influenza virus incursions of subtype H5N8, H5N5, H5N1, H5N4, and H5N3 in Germany during 2020–21. Virus Evol..

[46] Adlhoch C., Fusaro A., Gonzáles J., Kuiken T., Marangon S., Niqueux E., Staubach C., Terregino C., Baldinelli F. (2020). Avian influenza overview August–December 2020. EFSA J..

[47] Iancu I., Tirziu E., Pascu C., Costinar L., Degi J., Badea C., Gligor A., Bucur I., Popa S.A., Herman V. (2024). Evolution of HPAI avian influenza virus strains in Europe between 2005 and 2023. Rev. Rom. Med. Vet..

[48] Rosone F., Bonfante F., Sala M., Maniero S., Cersini A., Ricci I., Garofalo L., Caciolo D., Denisi A., Napolitan A. (2023). Seroconversion of a swine herd in a free-range rural multi-species farm against HPAI H5N1 2.3.4.4b clade virus. Microorganisms.

